# Elevated SGK1 increases Tau phosphorylation and microtubule instability in Alzheimer’s patient-derived cortical neurons

**DOI:** 10.1038/s41380-025-03225-4

**Published:** 2025-09-08

**Authors:** Komal Saleem, Zichun Xiao, Binglin Zhu, Yong Ren, Zhen Yan, Jian Feng

**Affiliations:** Department of Physiology and Biophysics, State University of New York at Buffalo, Buffalo, NY 14203 US

**Keywords:** Neuroscience, Stem cells

## Abstract

Hyperphosphorylation of Tau and the ensuing microtubule destabilization are linked to synaptic dysfunction in Alzheimer’s disease (AD). We find a marked increase of phosphorylated Tau (pTau) in cortical neurons differentiated from induced pluripotent stem cells (iPSCs) of AD patients. It is accompanied by significantly elevated expression of Serum and Glucocorticoid-regulated Kinase-1 (SGK1), which is induced by cellular stress, and Histone Deacetylase 6 (HDAC6), which deacetylates tubulin to destabilize microtubules. Indeed, acetylated tubulin and microtubule stability are significantly lower in AD-derived cortical neurons. SGK1 inhibitors or shRNA decrease Tau phosphorylation and HDAC6 levels while increasing acetylated tubulin in AD neurons. Overexpression of SGK1 in normal neurons does the opposite. These results suggest that elevation of the cellular stress-induced SGK1 increases Tau phosphorylation and HDAC6 expression, which destabilize microtubules to compromise many cellular functions subserving cognition. The coordinated increases in SGK1, pTau, and HDAC6, as well as the corresponding decrease in acetylated tubulin and microtubule stability in AD neurons, offer attractive targets for therapeutic development.

## Introduction

One of the pathological hallmarks of Alzheimer’s disease is the intracellular aggregates of hyperphosphorylated Tau, which show good correlation to cognitive decline [1, 2]. Tau hyperphosphorylation reduces its ability to stabilize microtubules [3–5] and increases its propensity to form aggregates [6]. Tau is phosphorylated by many kinases on up to 85 potential sites [7]. Recent phospho-proteome profiling of AD patient brains has identified dramatic changes in protein phosphorylation, with the microtubule-associated protein Tau as the most elevated phosphoprotein in AD [8]. Hyperphosphorylation of Tau is linked to synaptic dysfunctions and subsequent neurodegeneration in AD [9].

RNA sequencing (RNAseq) of the prefrontal cortex (PFC) of the P301S human Tau transgenic mouse model of dementia has identified Serum and Glucocorticoid-regulated Kinase-1 (*Sgk1*) as one of the top-ranking upregulated genes [10]. RT-qPCR experiments have confirmed the significantly higher level of *SGK1* mRNA in the PFC of AD human postmortem brain tissue [10]. Consistent with these, *SGK1* upregulation is observed in neurons from single-cell RNAseq of AD human frontal cortex [11] and neuron-specific RNAseq data from P301S mice [12]. Sgk1 plays a critical role in the formation and consolidation of spatial memory in the hippocampus [13]. Overexpression of a Sgk1 dominant-negative mutant worsens memory performance [14, 15]. Indeed, the selective SGK1 inhibitor GSK650394 decreases Tau hyperphosphorylation, restores synaptic transmission, and ameliorate memory deficits in P301S mice [10]. *SGK1* is an immediate early gene induced by a variety of cellular stress [16]. Among the many functions of SGK1, one direct link to AD is its ability to phosphorylate Tau [17], thus contributing to Tau hyperphosphorylation and microtubule depolymerization [18].

These evidence from AD mouse models and postmortem AD brains suggest that SGK1 has a critical role in Tau hyperphosphorylation and downstream cellular dysfunctions. It prompts us to examine iPSC-derived cortical neurons from AD patients to determine whether SGK1 is involved in Tau hyperphosphorylation. One of the significant hurdles in modeling AD with iPSC-derived neurons or NGN2-induced neurons for the past decade has been their inability to produce the 4R Tau isoform found in adult human brains [19–22]; only the fetal 0N3R splicing isoform is produced, even after one year of differentiation [19]. We have overcome this problem by installing the dorsal forebrain fate on neuralizing embryoid bodies to differentiate human iPSCs to cortical neurons expressing all six major splicing isoforms of Tau [23]. Using this method, we differentiated iPSCs from three normal subjects and three AD patients to cortical neurons. AD-derived cortical neurons exhibited marked increase of phosphorylated Tau (pTau) and SGK1. Inhibition or knockdown of SGK1 significantly decreased pTau in AD neurons, while SGK1 overexpression in control neurons increased Tau phosphorylation. Consistent with these, AD neurons showed a significant decrease in microtubule stability and acetylated tubulin (AcTub), and increased expression of HDAC6, a tubulin deacetylase [24]. SGK1 inhibition or knockdown significantly reduced HDAC6 and increased AcTub in AD neurons, while SGK1 overexpression did the opposite in control neurons.

## Results

### Increased Tau phosphorylation in iPSC-derived cortical neurons from AD patients

**Fig. 1:**
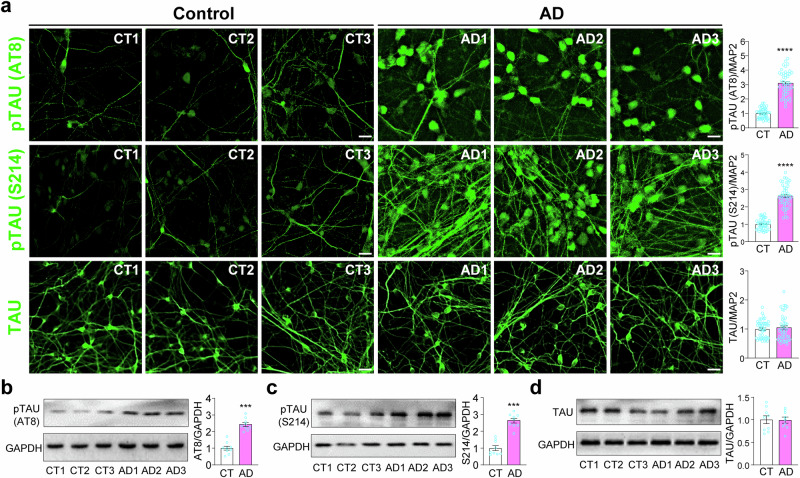
Increased Tau phosphorylation in iPSC-derived cortical neurons from AD patients. **a** Control or AD cortical neurons at day 40 were co-stained for MAP2, DAPI (Fig. S1), and phospho-specific Tau AT8 or S214, or total Tau. Relative fluorescence intensity of pTau (AT8), pTau (S214), and total Tau were quantified against MAP2 fluorescence in each sample. ****p < 0.0001, unpaired *t*-test, from 3 independent experiments of all 6 lines of neurons, each with at least 5 random frames. Bar, 50 µm. Data are presented as mean ± SEM. **b**–**d** Western blots of AT8 (b), S214 (c), or Tau (d) in total cell lysates were quantified against GAPDH. ***p < 0.001, unpaired *t*-test, from 3 independent experiments.

Using the method that we have developed [23], we differentiated three lines of control iPSCs and three lines of AD iPSCs to cortical neurons. All six lines of iPSCs were generated with the footprint-free plasmid method by the California Institute for Regenerative Medicine. Immunostaining of these iPSC-derived cortical neurons at Day 40 with the AT8 (for S202/T205 phosphorylation) [25] or S214 phospho-specific antibody against Tau showed a significant increase of pTau, while the total amount of Tau by immunostaining was very similar across the 6 lines (Fig. 1a), so was the level of MAP2 or DAPI costaining (Fig. S1). Western blotting of total cell lysates with the AT8 or S214 antibody confirmed the significant increase of pTau in AD-derived cortical neurons and the lack of change in total Tau (Fig. 1b–d).

### Elevated expression of SGK1 in cortical neurons derived from AD patients

**Fig. 2:**
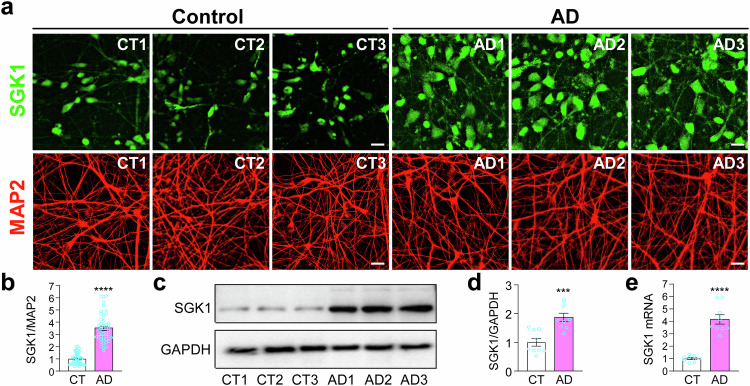
Increased SGK1 expression in iPSC-derived cortical neurons from AD patients. **a**, **b** Immunostaining of SGK1 and MAP2 in cortical neurons derived from unaffected control subjects and AD patients (a) and quantification of SGK1 fluorescence intensity against MAP2 intensity in each sample (b). **c**, **d** Western blots of SGK1 (c) in the total cell lysates were quantified against GAPDH (d). **e** RT-qPCR measurement of *SGK1* mRNA levels. ***p < 0.001, ****p < 0.0001, unpaired *t*-test, from 3 independent experiments, each with at least 5 random frames for (b). Bar, 50 µm.

As previous studies have shown increased expression of SGK1 in the P301S Tau transgenic mice and postmortem brain tissue from AD patients [10], we costained iPSC-derived cortical neurons from control subjects and AD patients for SGK1, MAP2, and DAPI (Fig. S2). There was a significant increase of SGK1 immunofluorescence (Fig. 2a, b), while MAP2 staining was very similar across the six lines of neurons (Fig. 2a). Western blotting of total cell lysates confirmed the significant increase in SGK1 protein levels (Fig. 2c, d). Consistent with these, *SGK1* mRNA levels were significantly elevated in RT-qPCR measurements (Fig. 2e). The increase in SGK1 expression and pTau levels were observed as early as day 25 (Fig. S3), when enough neurons could be produced for Western blotting as MAP2^+^ neurons were generated from day 18 in our method [23].

### SGK1 inhibition decreases Tau phosphorylation in AD cortical neurons

**Fig. 3:**
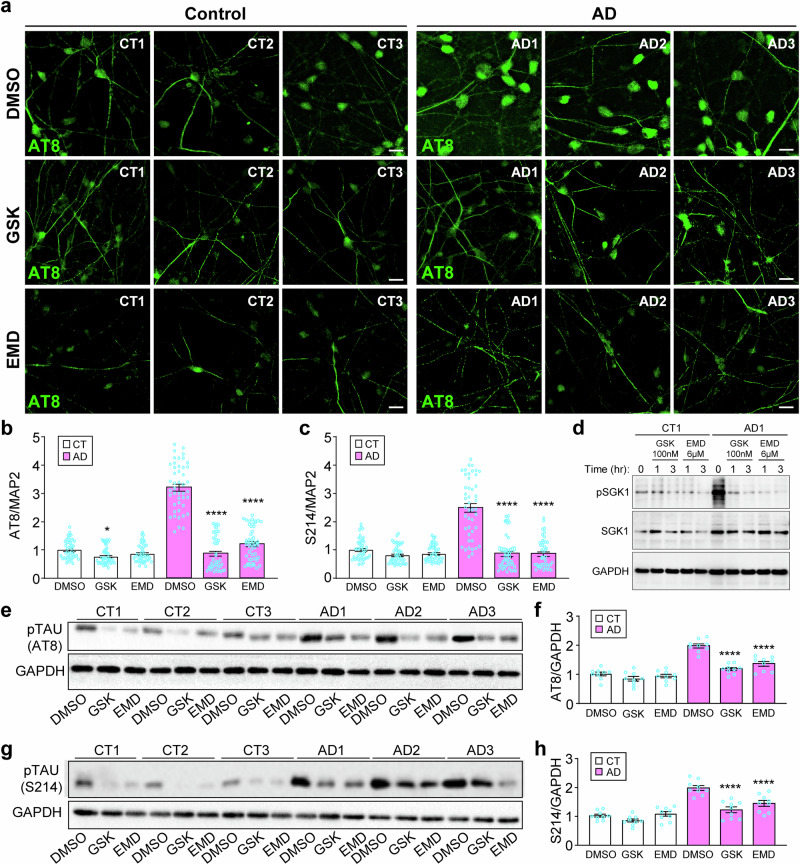
SGK1 inhibitors reduce Tau phosphorylation in cortical neurons derived from AD patients. **a**, **b** Control or AD cortical neurons treated with DMSO or the SGK1 inhibitors GSK650394 (100 nM) or EMD638683 (6 μM) for 72 h were costained for AT8 pTau (a), MAP2 and DAPI (Fig. S4). AT8 fluorescence was quantified against MAP2 fluorescence (b). **c** Quantification of S214 pTau fluorescence against MAP2 fluorescence (Fig. S5) in the same set of samples as in (a). **d** CT1 or AD1 neurons were treated and immunoblotted as indicated. **e**–**h** Western blots with AT8 (e) or S214 (g) antibodies of the same set of samples were quantified against GAPDH in (f) or (h), respectively. *p < 0.05, vs. DMSO for CT, ****p < 0.0001, vs. DMSO for AD, two-way ANOVA, from three independent experiments, each with at least 5 random frames for (b-c). Bar, 50 µm.

The increased expression of SGK1 and its ability to phosphorylate Tau [17] led us to examine whether the increased phosphorylation of Tau is connected to SGK1. We treated the 6 lines of cortical neurons with two different SGK1 inhibitors, GSK650394 (GSK, 100 nM) [26] or EMD638683 (6 µM) [27] for 72 h and found marked reduction in the immunostaining of AT8 or S214 phospho-specific antibodies (Figs. 3a–c, S4, S5). Western blotting of pSGK1, which recognizes activated SGK1 [28], confirmed the inhibition of activated SGK1 in AD neurons by GSK (100 nM) and EMD (6 µM). In contrast, control neurons had a much lower level of pSGK1 that was also decreased by GSK and EMD (Fig. 3d). We validated the immunostaining results above with Western blots (Fig. 3e–h). The reduction of Tau phosphorylation was much more pronounced in AD neurons than control neurons, which had a much lower pTau level at the basal condition (Fig. 3b, c, f, h).

### Manipulation of SGK1 expression consistently alters Tau phosphorylation

**Fig. 4:**
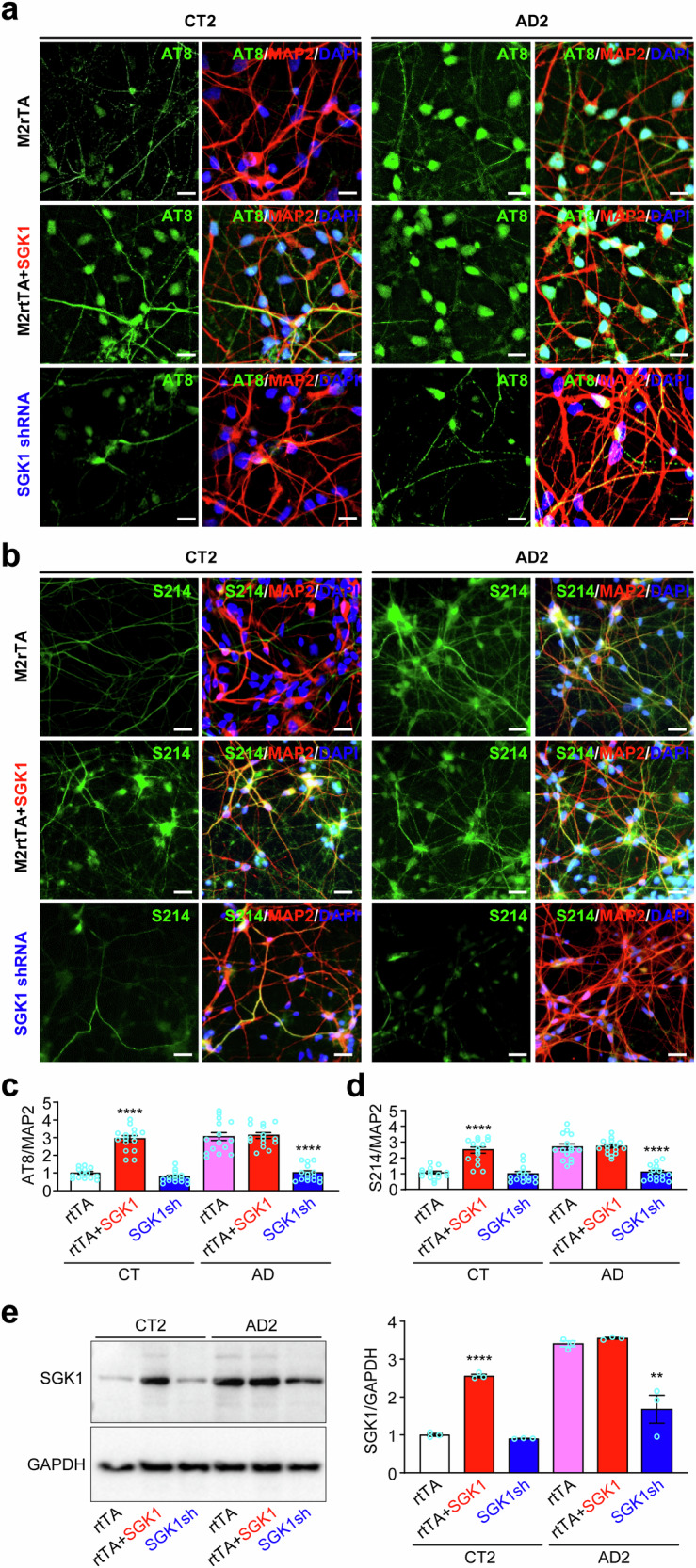
*SGK1* knockdown decreases pTau in AD neurons while SGK1 overexpression increases pTau in control neurons. **a**, **b** Control or AD cortical neurons infected with lentiviruses expressing *M2rtTA*, *M2rtTA* plus *SGK1*, or *SGK1* shRNA were costained for AT8 (a) or S214 (b) pTau, MAP2 and DAPI (Fig. S6). Representative images from CT2 and AD2 were shown. As M2rtTA and scrambled control shRNA had no significant effect in the experiment, we used M2rtTA as the control for both overexpression and knockdown of SGK1. **c**, **d** AT8 (c) or S214 (d) fluorescence intensity was quantified against MAP2 fluorescence intensity for each condition for the three control and three AD lines. **e** Western blotting and quantification of SGK1 in CT2 or AD2 neurons infected with these lentiviruses. **p < 0.01, ****p < 0.0001, vs. *M2rtTA*-transduced CT or AD, respectively, two-way ANOVA, from three independent experiments, each with at least 5 random frames for (c) and (d). Bar, 50 µm.

To substantiate the effects of the two different SGK1 inhibitors, we overexpressed SGK1 or knocked it down with lentivirus in the six lines of iPSC-derived cortical neurons and stained them with the AT8 or S214 phospho-specific antibodies (Figs. 4a, b, S6). SGK1 overexpression significantly increased Tau phosphorylation on S202/T205 (AT8 sites) and S214 in control neurons, but not beyond the already high levels in AD neurons (Fig. 4a, c). *SGK1* knockdown significantly decreased Tau phosphorylation on these sites in AD neurons, but not in control neurons, where pTau was already low (Fig. 4b, d). Western blotting showed that the doxycycline-inducible lentivirus of SGK1 significantly increased SGK1 expression in control neurons over its endogenous level, but did not significantly increase SGK1 expression in AD neurons beyond its already high endogenous level. Conversely, lentivirus expressing SGK1 shRNA significantly decreased the high endogenous SGK1 level in AD neurons, but not the low endogenous level in control neurons (Fig. 4e). Because lentiviruses expressing the reverse tetracycline transactivator M2rtTA or scrambled shRNA had no significant effect on SGK1 expression or pTau, we used M2rtTA as the transduction control.

### Decreased microtubule stability in cortical neurons derived from AD patients

**Fig. 5:**
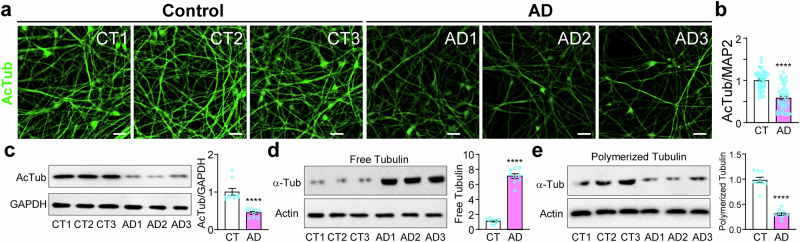
Decreased microtubule stability in cortical neurons derived from AD patients. **a**, **b** Immunostaining of acetylated tubulin (AcTub) in control and AD neurons (a) was quantified against MAP2 immunofluorescence (Fig. S7) (b). **c** Western blotting of AcTub in the same set of samples was quantified against GAPDH. **d**, **e** Western blotting of free tubulin (d) or polymerized tubulin (e) in the same samples were quantified against actin. ****p < 0.0001, vs. control neurons, unpaired *t*-test, from three independent experiments, each with at least 5 random frames for (b). Bar, 50 µm.

As Tau phosphorylation decreases its ability to bind to and stabilize microtubules [3–5], we examined microtubule stability in the six lines of cortical neurons with two independent methods. First, we costained the neurons for acetylated tubulin (AcTub) (Fig. 5a), a marker for stable microtubules [29], as well as MAP2 and DAPI (Fig. S7). AcTub fluorescence was significantly decreased in cortical neurons derived from AD patients (Fig. 5b). Western blotting of the total cell lysates confirmed the significant reduction of AcTub (Fig. 5c). Next, we measured the amount of free tubulin and polymerized tubulin (i.e. in microtubules) by lysing the cells at 37 °C to preserve microtubules [30]. The supernatant fraction of the total cell lysates contained the cytoplasm, including free tubulin, while the pellet fraction contained microtubules (i.e. polymerized tubulin). Western blotting showed significantly higher levels of free tubulin in the supernatant fraction (Fig. 5d) and significantly lower levels of polymerized tubulin in the pellet fraction from AD neurons (Fig. 5e).

### SGK1 knockdown increases acetylated tubulin in AD neurons while SGK1 overexpression decreases it in control neurons

**Fig. 6:**
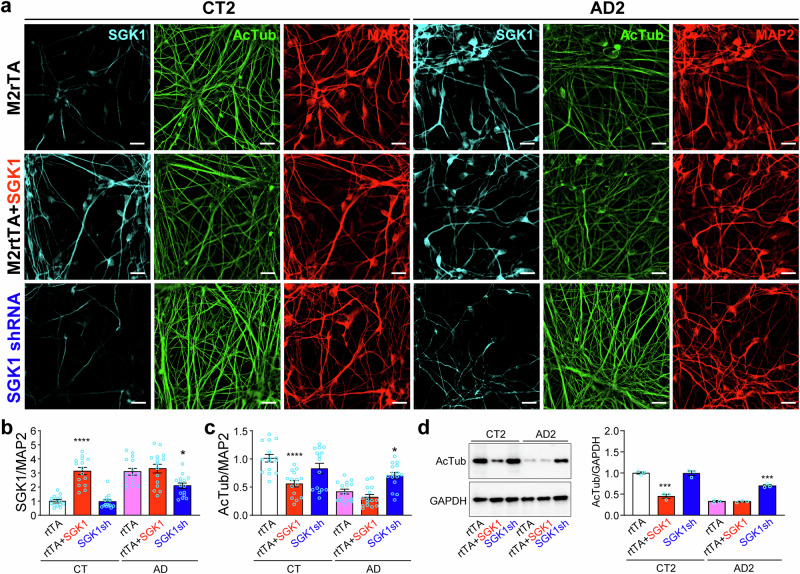
SGK1 overexpression decreases acetylated tubulin in control neurons while SGK1 knockdown increases acetylated tubulin in AD neurons. **a** Control and AD cortical neurons infected with lentiviruses expressing *M2rtTA*, *M2rtTA* plus *SGK1*, or *SGK1* shRNA were costained for SGK1, AcTub, and MAP2 (a). Representative images from CT2 and AD2 were shown. As M2rtTA and scrambled control shRNA had no significant effect in the experiment, we used M2rtTA as the control for both overexpression and knockdown of SGK1. **b**, **c** SGK1 (b) or AcTub (c) fluorescence was quantified against MAP2 fluorescence for the indicated conditions in the three control and three AD lines. **d** Western blotting and quantification of AcTub in CT2 and AD2 neurons transduced with the indicated lentiviruses. *p < 0.05, ***p < 0.001, ****p < 0.0001, vs. *M2rtTA*-transduced CT or AD, respectively, two-way ANOVA, from three independent experiments, each with at least 5 random frames for (b-c). Bar, 50 µm.

The increase in SGK1 and the decrease in AcTub in AD cortical neurons led us to examine whether SGK1 overexpression affects tubulin acetylation. When SGK1 was overexpressed (Figs. 4e, 6a, b), we found a significant decrease in AcTub in control neurons, but not AD neurons, by immunostaining (Fig. 6a, c) (Fig. S8) and Western blotting (Fig. 6d). When *SGK1* shRNA was overexpressed, there was a significant decrease of SGK1 in AD neurons, but not in control neurons (Figs. 4e, 6a, b), as the SGK1 level in control neurons was already very low. *SGK1* knockdown significantly increased AcTub in AD neurons, but not control neurons (Figs. 6a, c, d, S8).

### Increased expression of HDAC6 in cortical neurons derived from AD patients

**Fig. 7:**
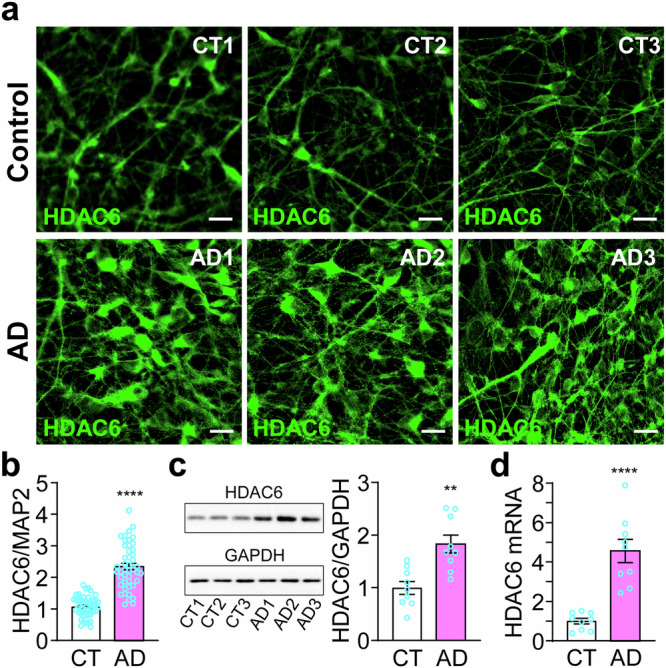
Increased expression of HDAC6 in cortical neurons derived from AD patients. **a**, **b** Immunostaining of HDAC6 in cortical neurons derived from normal subjects and AD patients (a) was quantified against MAP2 immunofluorescence (Fig. S9) (b). **c** Western blotting and quantification of HDAC6 in the same set of samples. **d** RT-qPCR measurement of *HDAC6* mRNA levels. **p < 0.01, ****p < 0.0001, vs. control neurons, unpaired *t*-test, from three independent experiments, each with at least 5 random frames for (b). Bar, 50 µm.

As HDAC6 is the main tubulin deacetylase [31, 32], the decreased AcTub level in AD cortical neurons prompted us to examine the expression of HDAC6 in these neurons. We found a significant increase of HDAC6 in cortical neurons derived from AD patients, as evidenced by immunostaining (Figs. 7a, b, S9) and Western blotting (Fig. 7c). Consistent with these, the amount of *HDAC6* mRNA was significantly increased (Fig. 7d).

### Manipulation of SGK1 consistently alters the expression of HDAC6

**Fig. 8:**
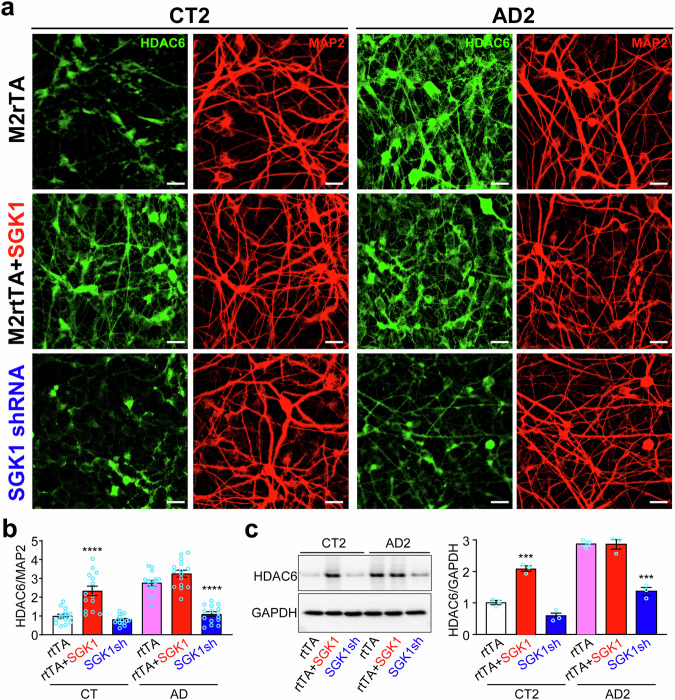
*SGK1* knockdown decreases HDAC6 in AD neurons while SGK1 overexpression increases HDAC6 in control neurons. **a** Control or AD cortical neurons infected with lentiviruses expressing *M2rtTA*, *M2rtTA* plus *SGK1*, or *SGK1* shRNA were costained for HDAC6 and MAP2. Representative images from CT2 and AD2 were shown. As M2rtTA and scrambled control shRNA had no significant effect in the experiment, we used M2rtTA as the control for both overexpression and knockdown of SGK1. **b** HDAC6 fluorescence was quantified against MAP2 fluorescence for each condition in the three control and three AD lines. **c** Western blotting and quantification of HDAC6 in CT2 and AD2 neurons transduced with the indicated lentiviruses. ***p < 0.001, ****p < 0.0001, vs. *M2rtTA*-transduced CT or AD, respectively, two-way ANOVA, from three independent experiments, each with at least 5 random frames for (b). Bar, 50 µm.

The increased expression of SGK1 (Fig. 2) and HDAC6 (Fig. 7) made us wonder whether they are connected. We overexpressed SGK1 and found a significant increase of HDAC6 in control neurons, but not in AD neurons, which already had a high level of HDAC6 (Figs. 8, S10). *SGK1* knockdown significantly decreased HDAC6 levels in AD neurons, but not appreciably in control neurons from their low HDAC6 levels (Figs. 8, S10).

### SGK1 inhibitors coordinately change SGK1, pTau, AcTub, and HDAC6 levels in AD neurons

**Fig. 9:**
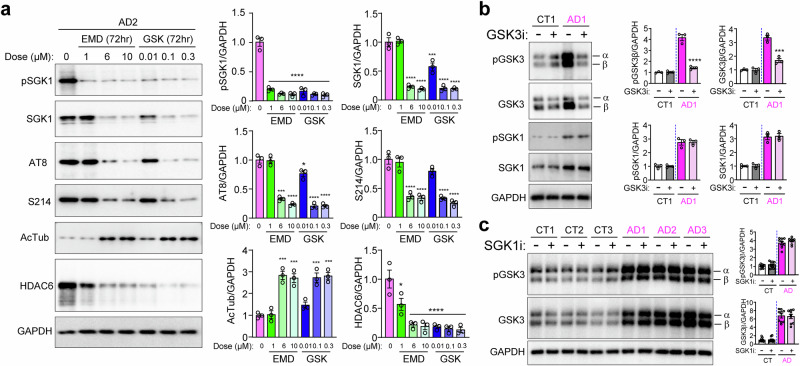
*SGK1* inhibition in AD neurons coordinately regulates SGK1, pTau, AcTub, and HDAC6 levels. **a** AD2 neurons were treated without or with the SGK1 inhibitor EMD638683 or GSK650394 for 72 h at the indicated concentrations. Western blots of the total cell lysates for phospho-SGK1, SGK1, AT8 or S214 pTau, AcTub, and HDAC6 were quantified against GAPDH. **b** CT1 and AD1 neurons were treated without or with the GSK3β inhibitor CHIR 99021 (1 µM for 48 h). Western blots of the total cell lysates for pGSK3, GSK3, pSGK1, and SGK1 were quantified against GAPDH. **c** Control and AD neurons were treated without or with the SGK1 inhibitor GSK650394 (100 nM for 72 h). Western blots of the total cell lysates for pGSK3 or GSK3 were quantified against GAPDH. *p < 0.05, **p < 0.01, ***p < 0.001, ****p < 0.0001, vs. 0 µM, two-way ANOVA for (a); vs. untreated AD1, unpaired t-test for (b), all from three experiments.

Results presented so far suggest that the changes of SGK1, pTau, AcTub, and HDAC6 in AD neurons may be connected. We treated AD2 neurons with the SGK1 inhibitor EMD638683 or GSK650394 for 72 h at increasing concentrations. Western blotting of the total cell lysates showed that the activated pSGK1 was inhibited by the inhibitors at all doses used (Fig. 9a). Interestingly, SGK1 level was decreased by each SGK1 inhibitor in a dose-dependent manner. Consistent with this, pTau levels at AT8 and S214 sites were reduced in parallel. There was a commensurate increase of acetylated tubulin and a corresponding decrease of HDAC6 (Fig. 9a).

To examine whether increased expression of GSK3β in AD [33] is connected to SGK1, we treated CT1 and AD1 neurons with the selective GSK3β inhibitor CHIR 99021 (1 µM for 48 h), which significantly inhibited the activated phospho-GSK3 (pGSK) α and β isoforms, and reduced the expression level of GSK3β in AD neurons (Fig. 9b). However, the GSK3β inhibitor did not affect the amount of activated pSGK1 or total SGK1 in AD neurons (Fig. 9b). The low basal levels of activated pGSK3α/β and pSGK1 in control neurons were not affected by CHIR 99021 (Fig. 9b). We also performed the reverse experiment by treating control and AD neurons with the SGK1 inhibitor GSK650394 (100 nM for 72 h) and found that it did not affect the level of activated pGSK3 (both α and β) and total GSK3 (both α and β) (Fig. 9c). These results suggest that GSK3β and SGK1 are independent of each other.

## Discussion

In this study, we have uncovered a coordinated series of cellular phenotypes in iPSC-derived cortical neurons from AD patients. A marked increase of pTau was accompanied by significantly higher expression of SGK1. When SGK1 was inhibited or knocked down in AD patient-derived neurons, pTau was significantly lowered, resembling the control condition. Conversely, SGK1 overexpression greatly increased pTau in control neurons, mimicking the situation in AD neurons. The SGK1-dependent increase of pTau parallels decreased AcTub and increased HDAC6, which are linked to reduced microtubule stability. Again, *SGK1* knockdown or inhibition in AD neurons reduced HDAC6 while SGK1 overexpression in control neurons increased HDAC6.

There are three important features about these phenotypes. First, all the changes have been found in postmortem brain tissue from AD patients and various mouse models of dementia. Increased pTau is a well-established pathogenic feature of AD [1, 2], so is the ensuing microtubule destabilization [3–5]. Elevated expression of SGK1 has been found in postmortem AD brains [10, 11] and the P301S human Tau transgenic mouse model of dementia [10, 12]. Expression of HDAC6 is increased in the cortex and hippocampus of AD patients [34–36]. HDAC6 inhibitors rescue cognitive dysfunction [37], restore microtubule-dependent axonal transport [38], and attenuate Tau-induced neurodegeneration [39] in AD mouse models by increasing the acetylation of tubulin [24] and Tau [40]. Increased AcTub stabilizes microtubules and promotes microtubule-based transport [41], while increased Tau acetylation reduces Tau phosphorylation [34, 42].

Second, the increase in SGK1, HDAC6, and pTau, as well as the decrease in AcTub and microtubule stability appear to be interconnected and may reflect elevated cellular stress in AD. SGK1 is induced by oxidative stress [43–45], which is substantially elevated in AD through a variety of mechanisms [46]. As a cellular stress-induced kinase [16], SGK1 phosphorylates many proteins, including Tau [17]. Indeed, this study showed for the first time that SGK1 inhibition or knockdown reduced Tau phosphorylation in cortical neurons, and more prominently in those from AD patients than control subjects. On the other hand, SGK1 overexpression markedly increased pTau in control neurons, but not AD neurons. SGK1 appears to function as a homeostatic regulator that senses cellular stress levels and mobilizes a counteracting response when necessary. In the AD state, elevated oxidative stress may induce SGK1 to increase pTau, which destabilizes microtubules and attenuates microtubule-dependent transport. This would reduce neurotransmission [47] and the requisite ATP production through mitochondrial respiration, which generates reactive oxygen species. Thus, overexpression of SGK1 in AD neurons failed to increase pTau further, while SGK1 knockdown or inhibition in control neurons had very little effect on the already low level of pTau. As SGK1 also regulated HDAC6 levels in a homeostatic manner, elevated HDAC6 in AD neurons reduced AcTub, which destabilize microtubules to reduce neurotransmission [47]. Future studies will understand the mechanistic details and the exact sequence of events regarding the five interconnected phenotypes we uncovered in AD neurons: elevated SGK1, pTau, and HDAC6 levels and the corresponding decrease in AcTub and microtubule stability.

Third, all these phenotypes have critical roles in AD pathogenic mechanisms. Tau hyperphosphorylation, which triggers the formation of neurofibrillary tangles, is a key pathological hallmark of AD [48]. Elevated SGK1 directly contributed to Tau hyperphosphorylation as evidenced by our results. SGK1 inhibition lowers pTau and rescues the deficits in synaptic transmission and memory tests in P301S mice [10]. Increased expression of HDAC6 decreases Tau acetylation [40], which is a key factor contributing to the formation of Tau aggregates [39, 49]. In addition, phosphorylation of Tau reduces its ability to bind and stabilize microtubules [3–5]. The reduction of AcTub induced by increased expression of HDAC6 further destabilizes microtubules. The combined effects of SGK1 and HDAC6 on microtubule destabilization would significantly attenuate microtubule-based transport, on which synaptic transmission relies [47, 50].

SGK1 phosphorylates many substrates in different types of cells [16]. For example, it phosphorylates the transcription factor FOXO3a to inhibit its nuclear translocation and transcriptional activity, thus suppressing apoptosis and promoting cell survival [51]. SGK1 also phosphorylates HDAC4 to regulate epigenetic control in the transcription of many genes [52]. By phosphorylating the ubiquitin ligase NEDD4-2, SGK1 regulates the cell surface expression of ENaC channels [53]. As SGK1 appears to exert a broad and coordinated homeostatic response to maximize cell survival at different conditions, inhibition of SGK1, which also reduced its expression, seems to be an attractive strategy for therapeutic development in AD. Both SGK1 and HDAC6 are expressed in non-neurons as well as in neurons. Various methods have been developed to differentiate human iPSCs to microglia, astrocytes, oligodendrocytes, and endothelial cells of blood vessels in the brain [54, 55]. It would be useful to develop a mixed culture system to enable the examination of SGK1 and HDAC6 in the complex interaction of neurons with these non-neuronal cells. The availability of iPSC-derived cortical neurons from AD patients and the discovery of SGK1 and HDAC6 as key targets based on pathogenic mechanisms would stimulate further research and drug discovery in Alzheimer’s disease.

## Methods

### Ethics approval and consent to participate

The use of CF-1 mice to produce mouse embryonic fibroblasts for culturing human iPSCs is approved by the University at Buffalo Institutional Animal Care and Use Committee. The use of human iPSCs from de-identified commercial sources is not human subject research. All methods used in the study were performed in accordance with guidelines of International Society for Stem Cell Research.

### Human pluripotent stem cells

Six lines of human iPSC (hiPSC) were purchased from the Human Pluripotent Stem Cell Line Repository of California Institute for Regenerative Medicine. Three lines are generated from healthy control subjects [CW70305 (female, 56 years), CW70344 (male, 62 years), CW50040 (male, 63 years)] and three are from patients with AD [CW50018 (female, 58 years), CW50024 (male, 63 years, CW50170 (female, 64 years)]. CIRM does not provide specific details on the clinical diagnosis of AD and the presence or absence of family history or disease-causing genetic mutations on these patients. As AD-causing monogenic mutations are present in less than 1% of AD cases [56] and definitive diagnosis of AD requires neuropathology of the postmortem brain, a limitation of the study is that the patients only have a clinical diagnosis of AD. We selected early-onset AD patients with the assumption that these patients may have stronger inborn vulnerabilities that can be embodied in iPSC-derived cortical neurons. All six lines are made with non-integrating episomal vectors expressing Yamanaka factors. The hiPSCs cells were cultured on gamma-irradiated CF-1 mouse embryonic fibroblasts in a medium containing DMEM/F12, 20% knockout serum replacement, 0.1 mM β-mercaptoethanol, 1× NEAA, 1× L-glutamine, and 4–8 ng/mL FGF2. The medium was changed daily. Cells were passaged with dispase (1 mg/ml), washed, and replated at a ratio of 1:6 weekly. Periodical mycoplasma tests by PCR showed no contamination.

### Differentiation of hiPSCs to cortex neurons

The human iPSCs were differentiated to cortical neurons as described [23]. Briefly, hiPSCs were dissociated with dispase to generate embryoid bodies (EBs), which were cultured in suspension in a 1:1 mixture of DMEM/F12 and Neurobasal media with N2 Supplements (1:100), B27 supplements without vitamin A (1:50), 1X NEAA, ascorbic acid (0.2 mM), SB431542 (10 µM, days 0–6), dorsomorphin dihydrochloride (5 µM, days 0–6), XAV939 (2.5 μM, days 0–10) and Cyclopamine (3.5 μM, days 0–10). On day 6, the EBs were plated on Matrigel-coated plates and cultured in the same media without SB431542 and dorsomorphin, with the medium changed every other day. XAV939 and Cyclopamine were stopped on day 10. On day 12, specified dorsal forebrain neuroepithelial cells in rosettes were dissociated into single cells with 1 unit/ml Accutase at 37 °C for 5 min and plated onto polyornithine/Matrigel-coated plates at a density of 5000–10,000 cells/cm^2^ in a 1:1 medium of DMEM/F12 and Neurobasal that contained N2, B27 without vitamin A (1:50), 1X NEAA and ascorbic acid (0.2 mM). The ROCK inhibitor Y27632 (20 μM) was added during the first 24 h. At day 18, cells were dissociated and plated in the Neurobasal medium that contained B27 without vitamin A (1:50), Brain-derived Neurotrophic Factor (BDNF) (20 ng/ml), Glial cell line-derived Neurotrophic Factor (GDNF) (20 ng/ml), dibutyryl-cAMP (dcAMP) (0.25 mM) and DAPT (2.5 μM). Half of the medium was changed every other day. MAP2^+^ neurons appeared from day 18. All experiments were performed on day 40 unless described otherwise.

### Generation of SGK1 and SGK1 shRNA lentiviruses

Human *SGK1* ORF was purchased from Addgene (#83432) and subcloned into the FUW-tetO-LoxP lentiviral vector [57]. FUW-M2rtTA (M2 reverse tetracycline-controlled transactivator) was purchased from Addgene (#20342). *SGK1* shRNA sequence (CTGGAAGCTTAGCAATCTTAT) was selected from the TRC (RNAi Consortium) shRNA library (TRCN0000194957) and cloned into the pLKO.1 vector. pLKO.1-Scrambled control shRNA vector was purchased from Addgene (#136035). All constructs made by us were verified by sequencing. HEK293FT cells (2.5 × 10^6^ in a 10 cm dish) were transfected with 10 µg lentiviral transfer vector, 2.5 µg pMD2.G envelope plasmid, and 7.5 µg psPAX2 packaging plasmid in OptiMEM (Gibco, 31985) with Lipofectamine 2000 according to the manufacturer’s instructions (Invitrogen, 11668-019). Media containing the lentivirus was collected at 24, 36 and 48 hr post-transfection. Pooled virus was titered using a p24 ELISA kit (Xpress BIO, XB-1000). Control and AD cortical neurons infected with lentiviruses expressing M2rtTA and SGK1 were treated with doxycycline (1 µg/ml for 48 h) to overexpress SGK1 or with lentivirus expressing SGK1 or scrambled shRNA for 48 h to knockdown SGK1. As M2rtTA and scrambled control shRNA had no significant effect in the experiments, we used M2rtTA as the control for both overexpression and knockdown of SGK1.

### Real-time quantitative PCR

Total RNA was extracted from iPSC-derived neuronal cultures at D40 using TRIzol (ThermoFisher) and cleaned with an RNeasy kit (QIAGEN). After 1.0 μg total RNA was reverse transcribed to cDNA with the iScript^TM^ cDNA synthesis kit (BIO-RAD), qPCR was performed with IQ SYBR Green Supermix (BIO-RAD). Each cDNA was run in triplicate. PCR conditions included an initial denaturation step of 4 min at 95 C, followed by 40 cycles of PCR consisting of 30 s at 95 °C, 30 s at 60 °C and 60 s at 72 °C. Average threshold cycle (Ct) values from the triplicate PCR reactions for a gene of interest were normalized against the average Ct values for *GAPDH* from the same cDNA sample. All results are from three technical replicates of three independent biological samples for each subject. PCR primers for *SGK1* were TGGGCTACCTGCATTCACTG and CTCAGGTGCGAGATACTCCG, for *HDAC6* were GCCAGAAACTTGGTGGAGCG and ACTGGGGGTTCTGCCTACTT, and for *GAPDH* were AGATCCCTCCAAAATCAAGT and CAGAGATGATGACCCTTTTG.

### Immunocytochemistry

Cells cultured on glass coverslips in 12-well plates were fixed with 4% paraformaldehyde in PBS for 20 min, permeabilized with 0.1% Triton X-100 in PBS with Ca^2+^/Mg^2+^ for 20 min at room temperature and blocked in 3% BSA in PBS with Ca^2+^/Mg^2+^ for 60 min at room temperature. Cells were incubated in primary antibody in 3% BSA in PBS with Ca^2+^/Mg^2+^ at 4 °C overnight, washed with PBS with Ca^2+^/Mg^2+^ for three times, then incubated with secondary antibody in 3% BSA in PBS with Ca^2+^/Mg^2+^ at room temperature for 2 h. The nucleus was labeled with 1 μg/ml DAPI for 10 min at room temperature. Images were collected with a Leica AF6000 inverted fluorescence microscope with a CMOS camera. Integrated fluorescence intensity with background subtraction was calculated using NIH Image J for each image. At least 5 random images were quantified for a line of neurons at each condition.

### Western blot

Cells were washed three times in cold PBS buffer and lysed in 1% SDS with protease inhibitor for 10 min at room temperature. Lysates were boiled for 5 min at 100 °C and centrifuged at 13,000 g for 10 min. The supernatant was separated on sodium dodecyl sulfate (SDS)–polyacrylamide gels and analyzed by Western blotting with antibodies against SGK1 (SAB, 32125), AT8 (Thermo Fisher, MN1020), S214 (Thermo Fisher, 44-742G), TAU (Thermo Fisher, AHB0042), Ac-tubulin (Proteintech,66200-1-Ig), HDAC6 (Proteintech, 12834-1-AP), or MAP2 (Santa Cruz, SC-6260). ECL detection was performed according to the manufacturer’s protocol (Amersham, Piscataway, NJ).

### Measurement of free or polymerized tubulin in the cell

Free or polymerized tubulin from iPSC-derived neuronal cultures was extracted using a protocol described previously [30]. Briefly, neuronal cultures at D40 were washed twice at 37 °C with 1 ml of Buffer A containing 0.1 M MES, pH 6.75, 1 mM MgSO_4_, 2 mM EGTA, 0.1 mM EDTA, and 4 M glycerol. After the neuronal cultures were incubated at 37 °C for 5 min in 600 µl of free tubulin extraction buffer (Buffer A plus 0.1% v/v Triton X-100 and protease inhibitors), the extracts were centrifuged at 37 °C for 2 min at 16,000 × g. The supernatant fractions contained free tubulin extracted from the cytosol. The pellet fraction and lysed cells in the culture dish were dissolved in 600 µl of 25 mM Tris, pH 6.8, plus 0.5% SDS. This fraction contained tubulin originally in a polymerized state (i.e. as microtubules). Equal amounts of total proteins from free and polymerized tubulin fractions were analyzed by Western blotting with anti-tubulin antibody (Sigma). The intensity of tubulin bands was quantified against actin bands from three different experiments with NIH Image J.

### Statistical analysis

All statistical analysis was performed using GraphPad Prism 6. All data are expressed as mean ± standard error of measurement (SEM). Unpaired student *t*-test was used for comparing two groups and two-way ANOVA was used for comparing multiple groups with different treatments. Sample size is based on the observed effect size in preliminary studies.

## Supplementary information

Supplemental Figures 1-10 and Supplemental Table 1

## Data Availability

All data are included in the manuscript, Supplementary Figures, and the Supplementary Table.
