# Supplementary material for: Elevated SGK1 increases Tau phosphorylation and microtubule instability in Alzheimer’s patient-derived cortical neurons
Source: Mol Psychiatry. 2025 Sep 8;31(1):332–42. doi: 10.1038/s41380-025-03225-4 (PMC12700806; doi:10.1038/s41380-025-03225-4)
Supplement: Supplementary file 1 — Supplemental Figures 1-10 and Supplemental Table 1 [file 41380_2025_3225_MOESM1_ESM.pdf]

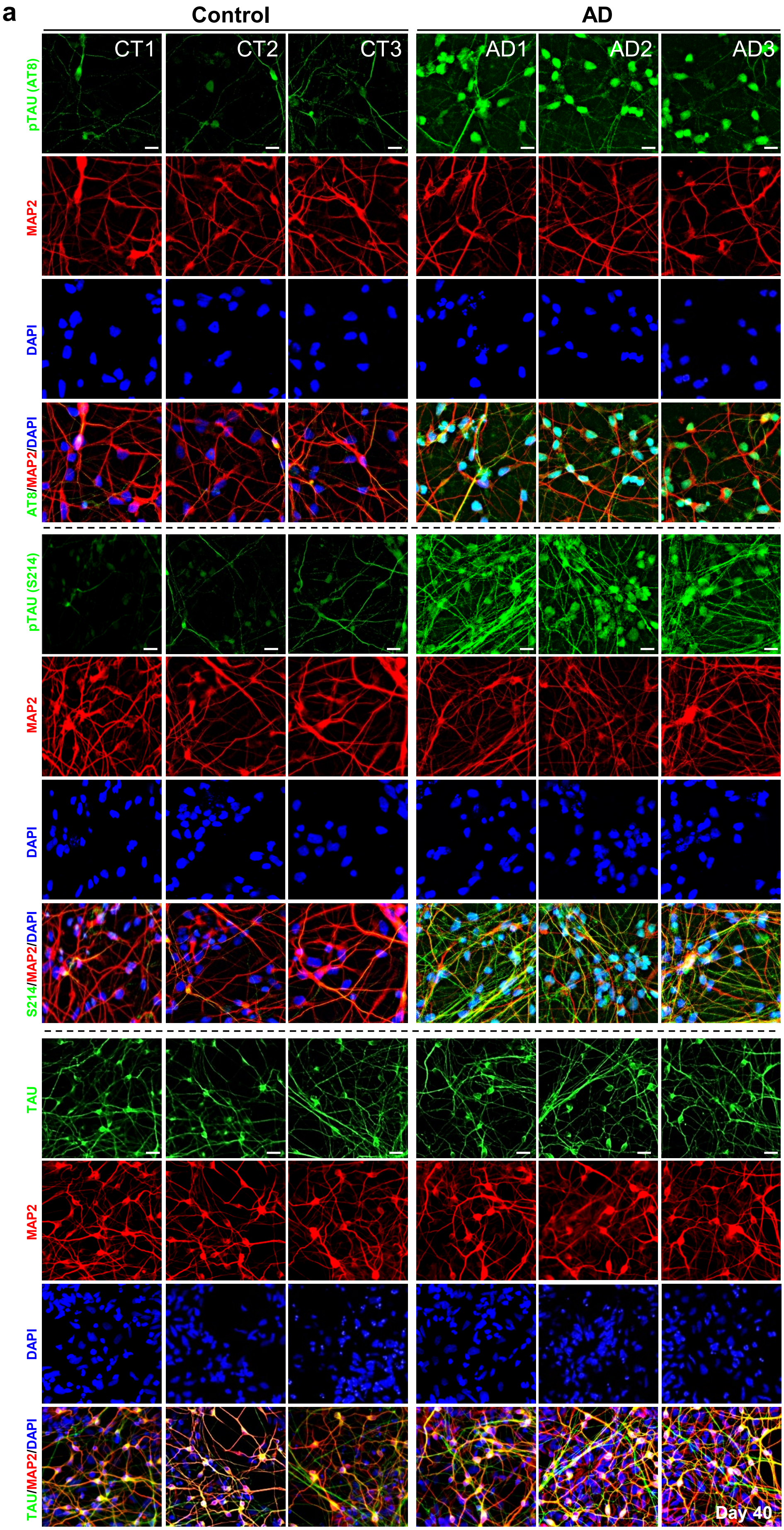

Supplementary Fig. S1. Separate channels of Fig. 1a

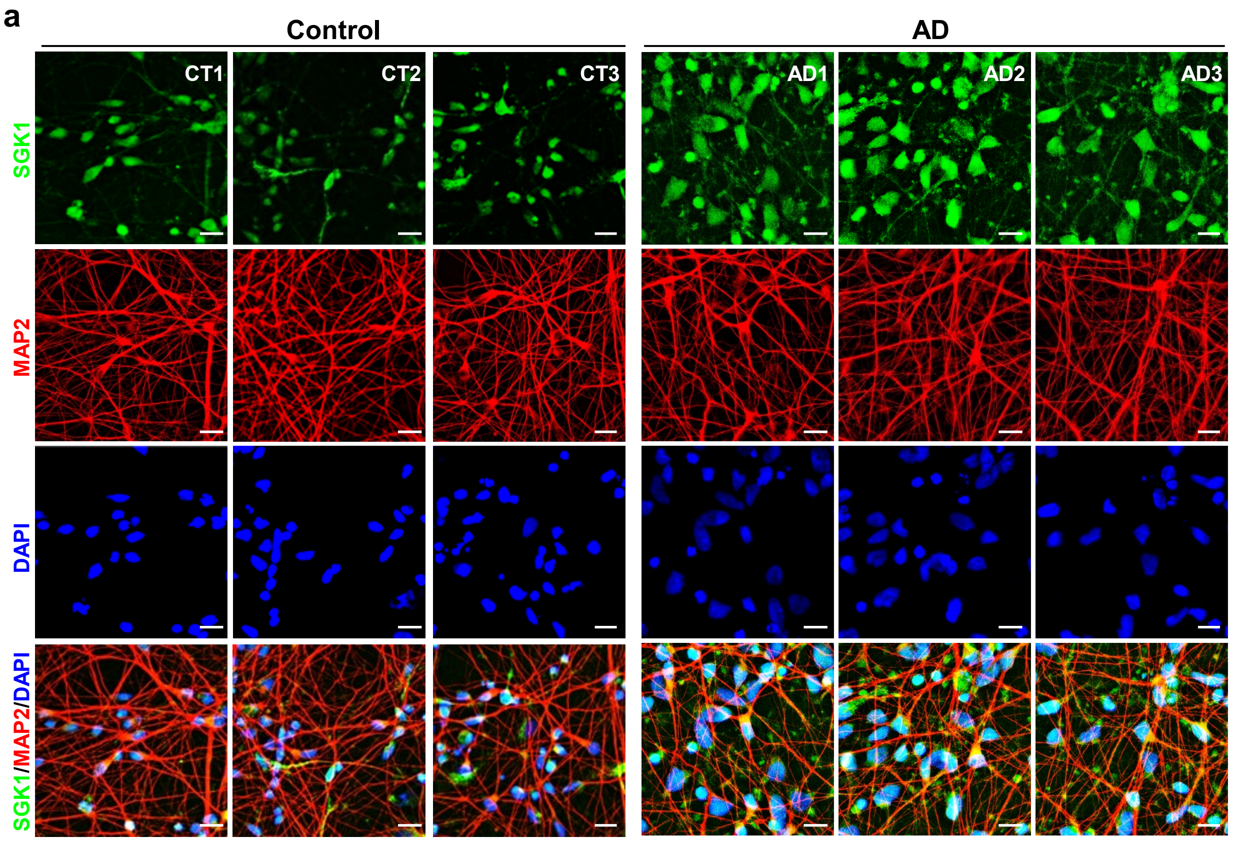

Supplementary Fig. S2. Separate channels of Fig. 2a

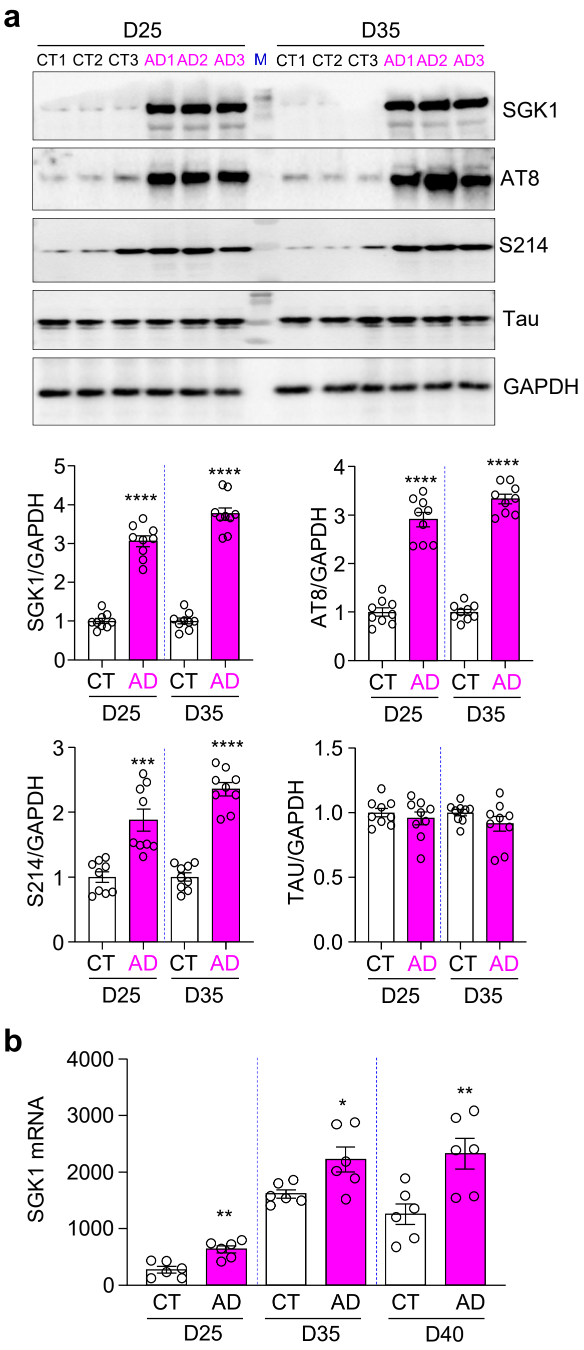

Supplementary Fig. S3. Increased SGK1 expression and Tau phosphorylation in AD neurons at the early stage of neuronal differentiation. a, Western blotting and quantification of SGK1, AT8 and S214 pTau, and Tau in the total cell lysates of control and AD neurons at days 25 and 35 of differentiation. M, prestained molecular weight markers. $MAP2^+$ neurons were generated from specified dorsal forebrain neuroepithelial cells starting from day 18. **b**, RT-qPCR measurement of SGK1 mRNA in the three control and three AD lines of neurons at the indicated time points. *p<0.05, **p<0.01, ***p<0.001, ****p<0.0001, unpaired *t*-test, vs. CT at the same time point, from three experiments.

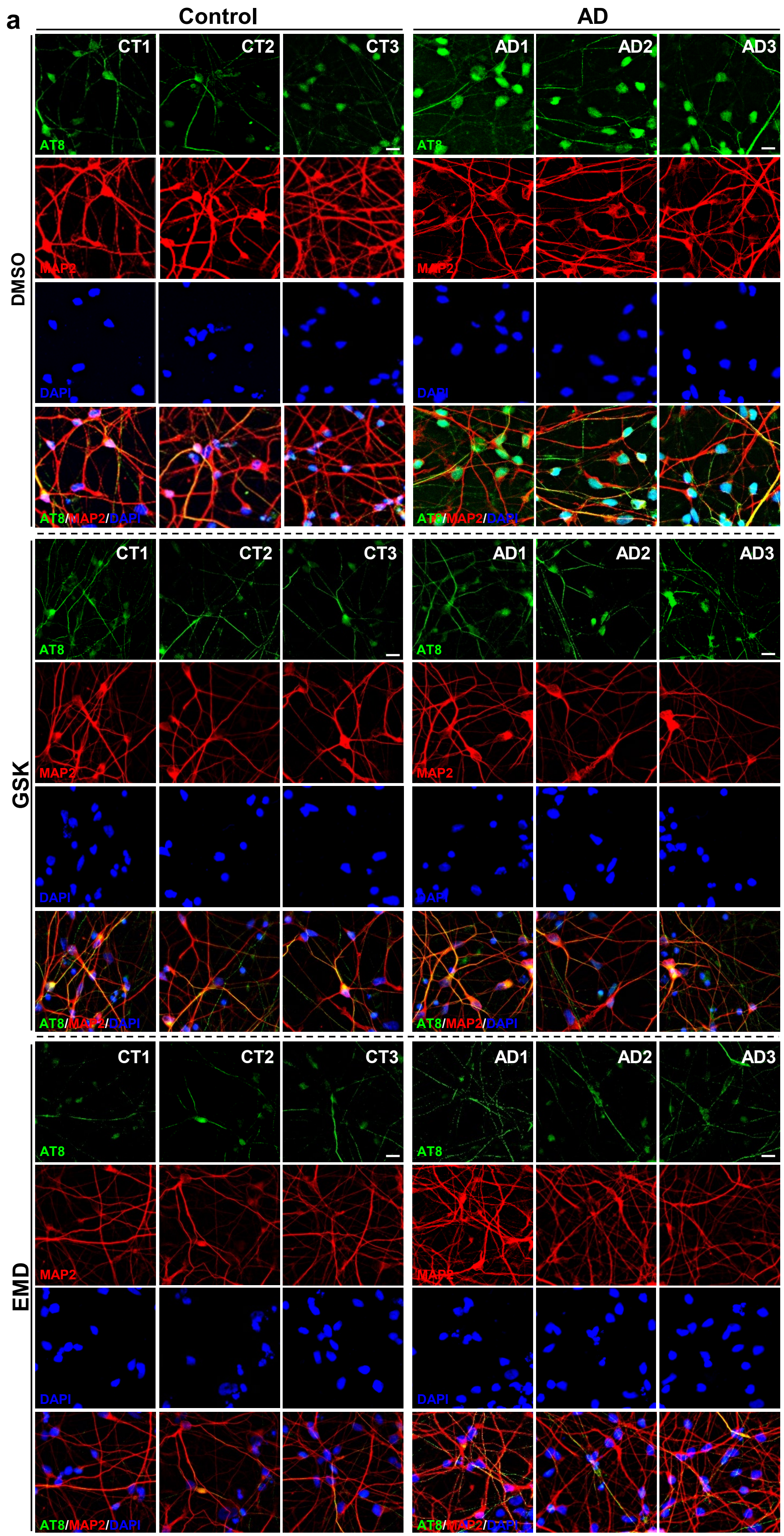

Supplementary Fig. S4. Separate channels of Fig. 3a

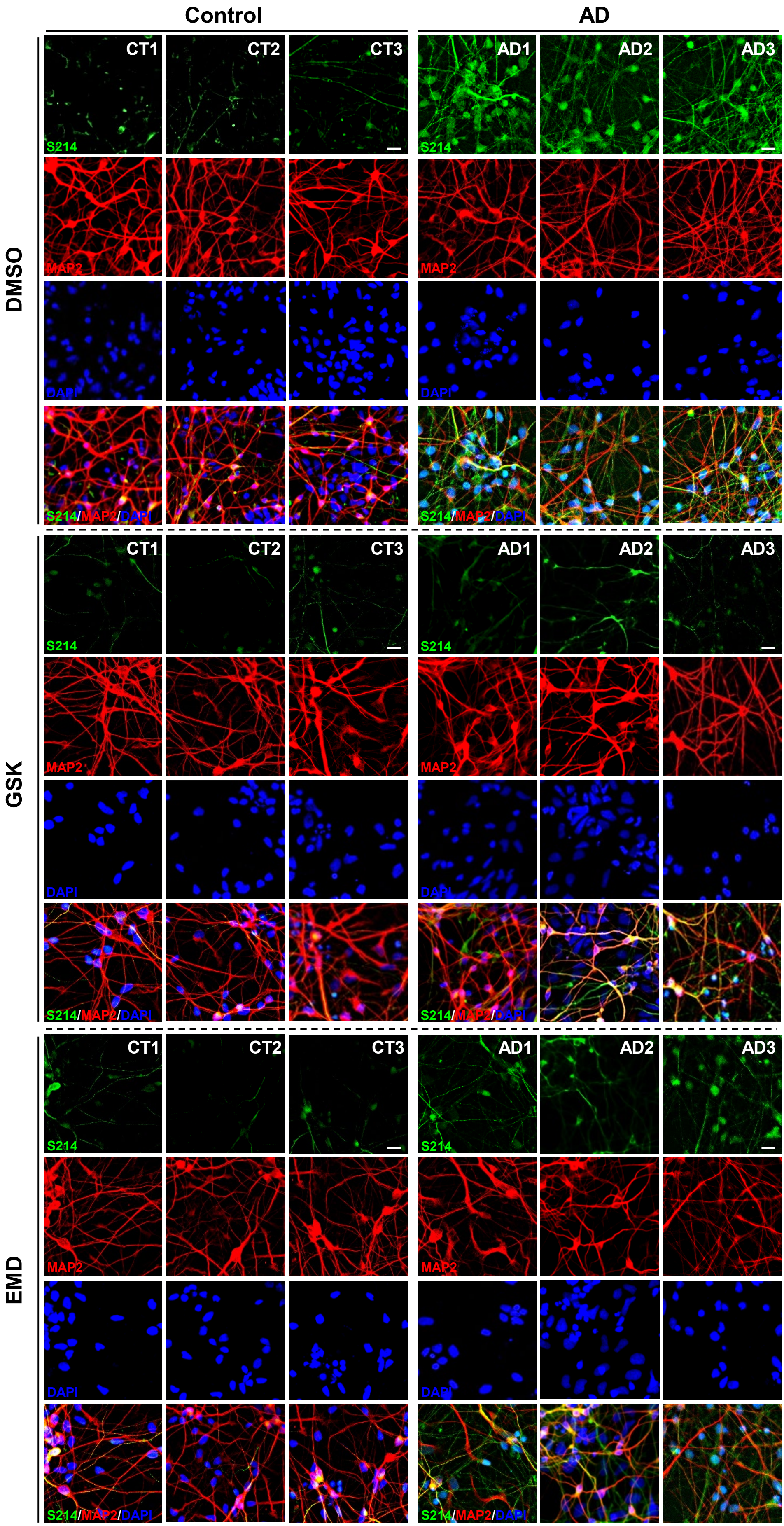

Supplementary Fig. S5. The effect of SGK1 inhibitors on Tau phosphorylation at S214 (for Fig. 3c)

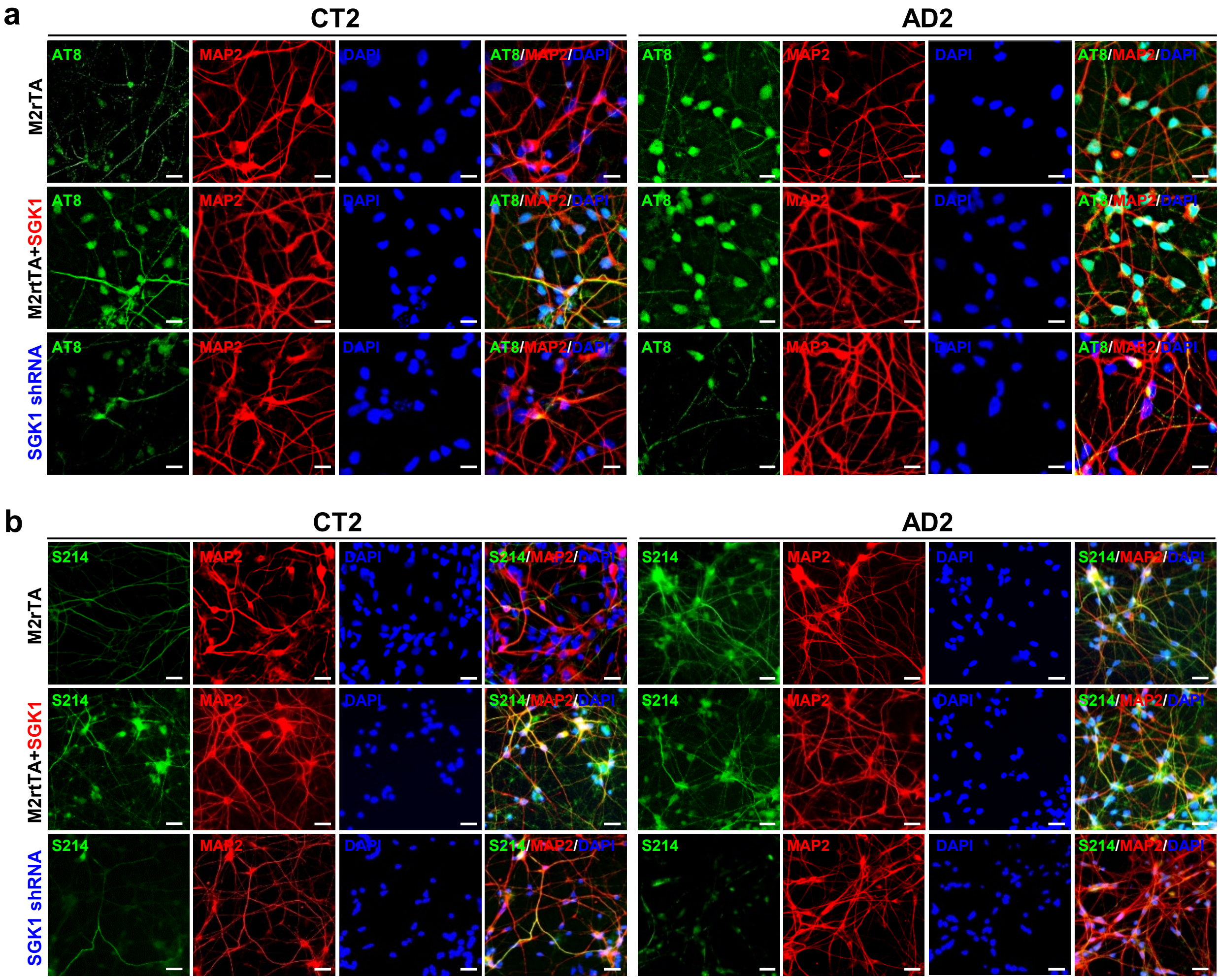

Supplementary Fig. S6. Separate channels of Fig. 4a and Fig. 4b.

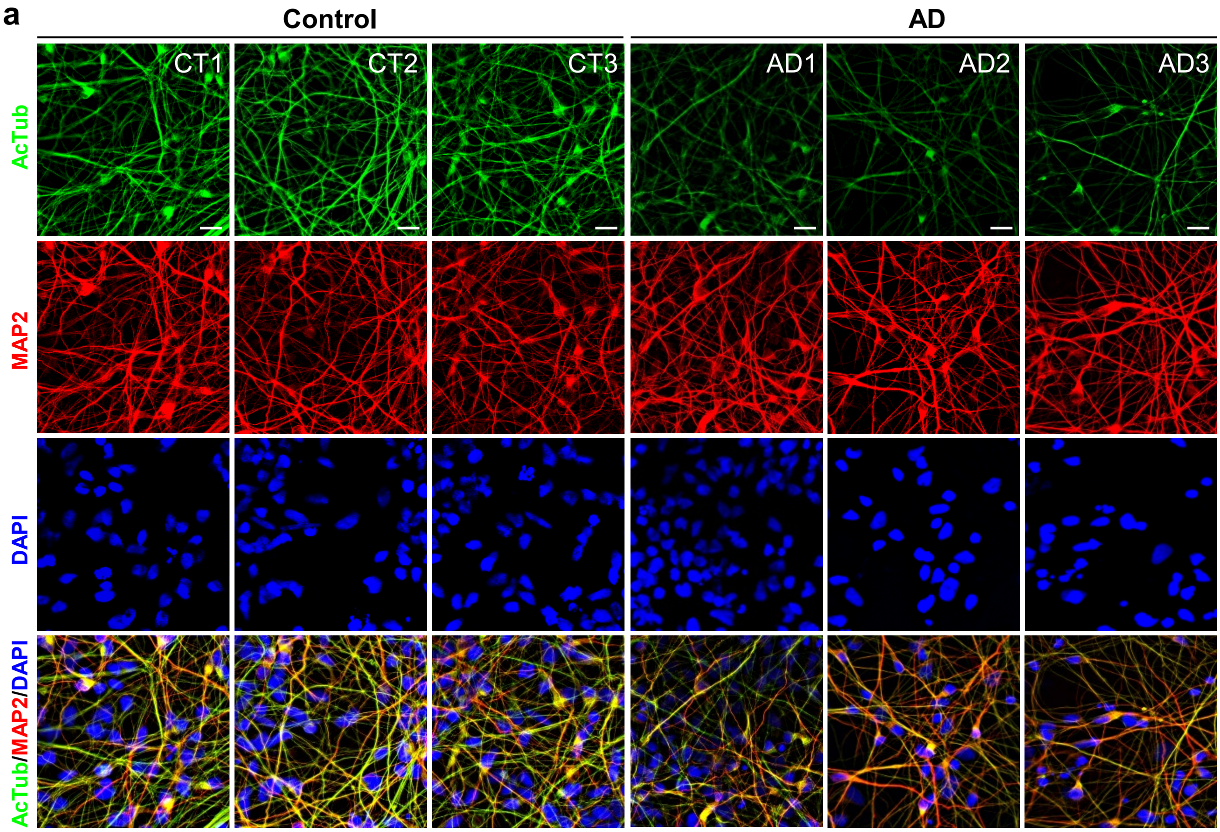

Supplementary Fig. S7. Separate channels of Fig. 5a

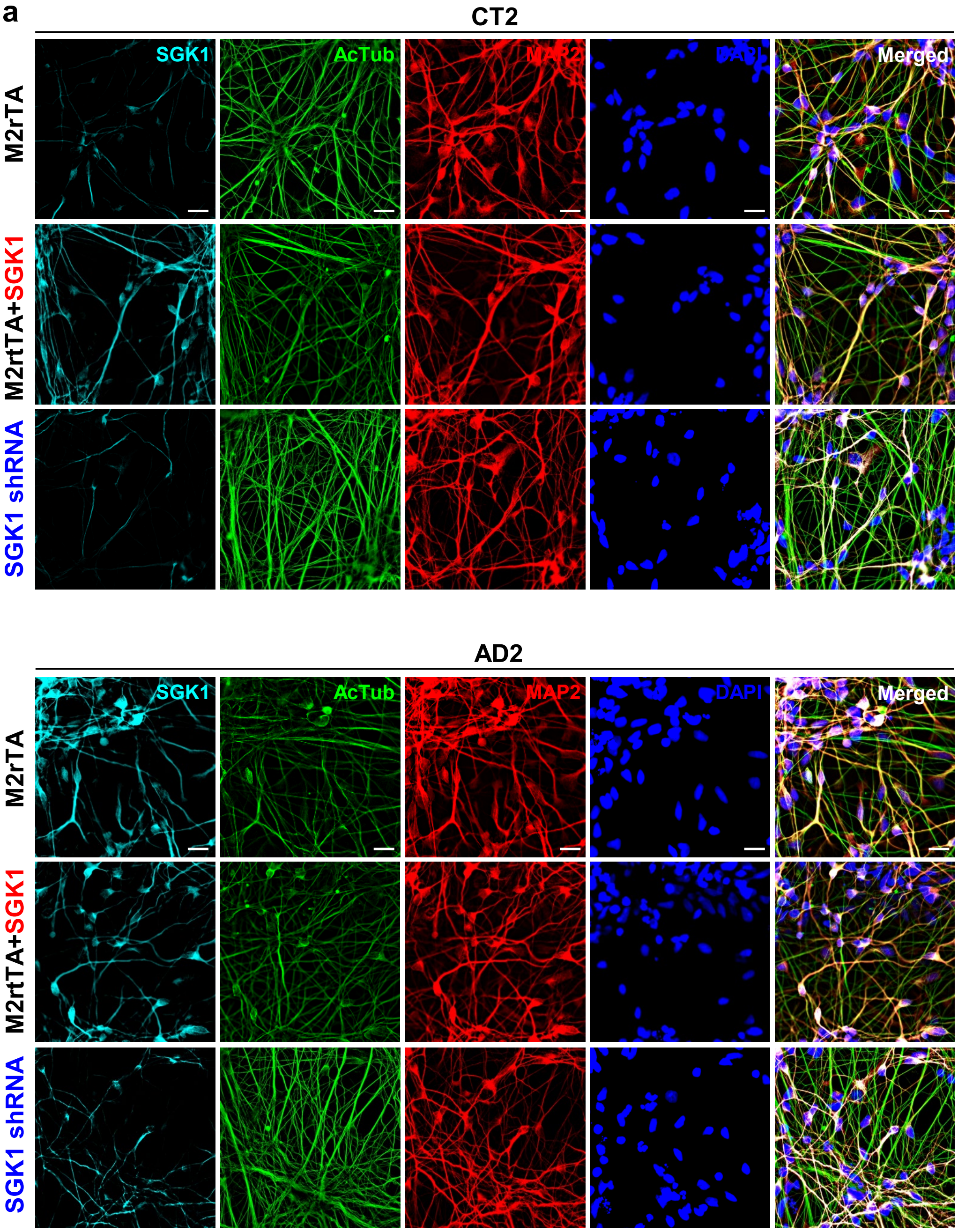

Supplementary Fig. S8. Separate channels of Fig. 6a.

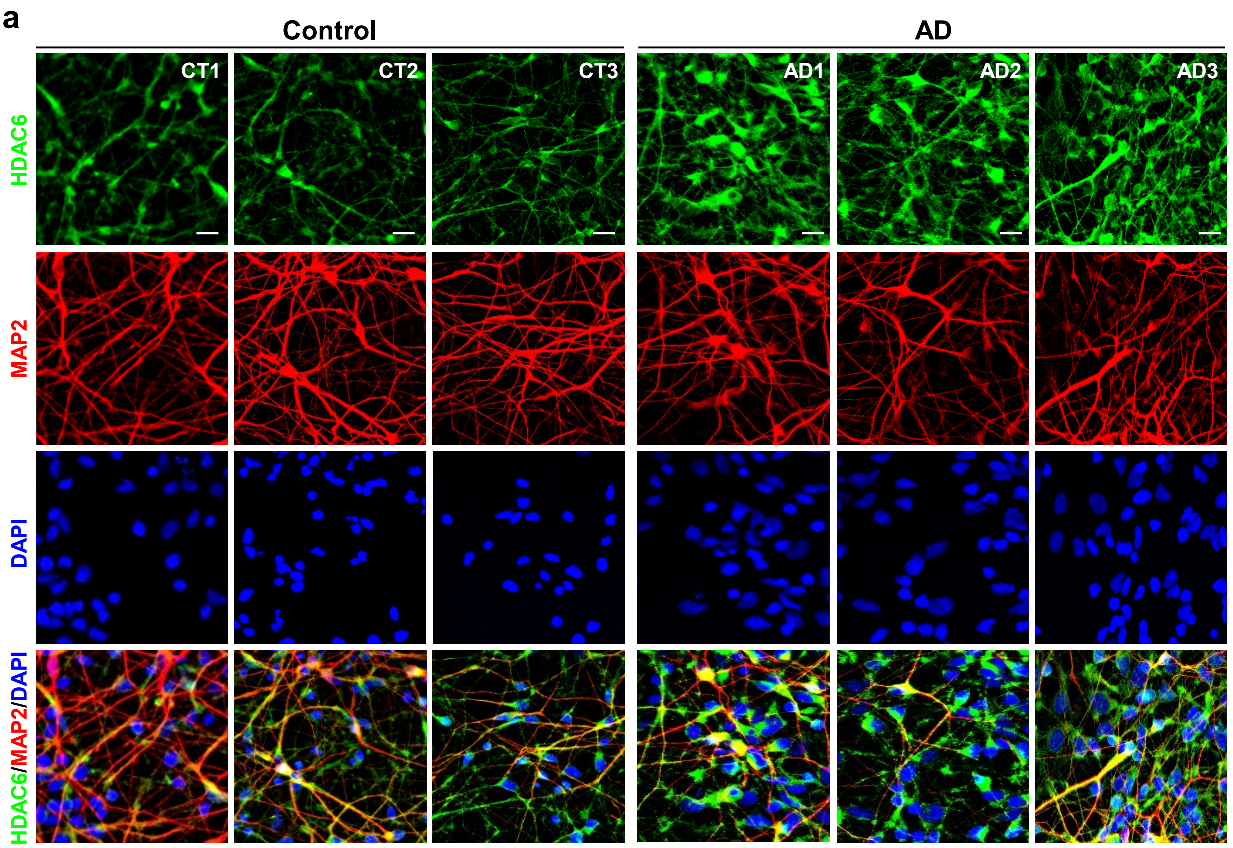

Supplementary Fig. S9. Separate channels of Fig. 7a

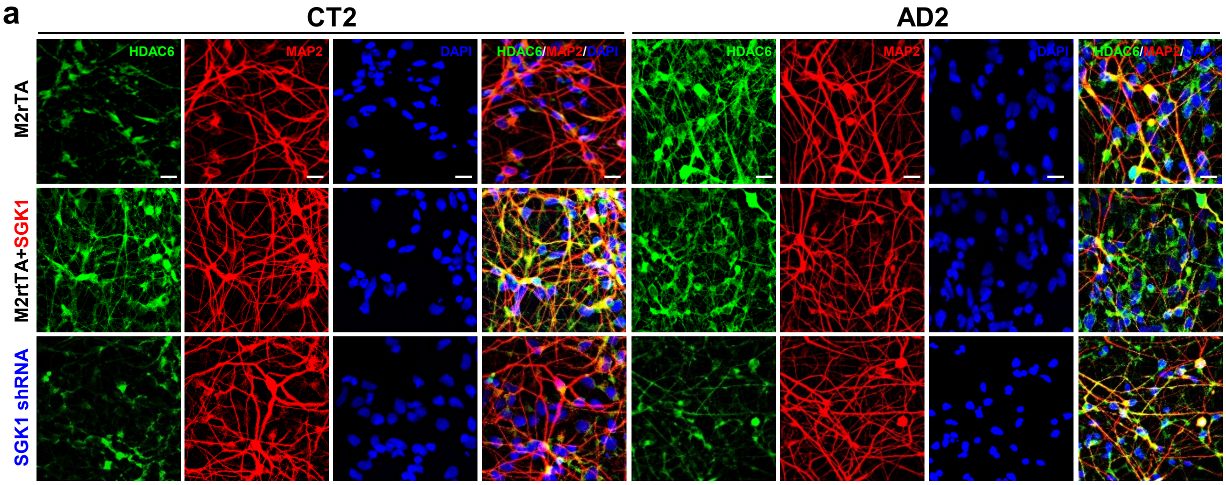

Supplementary Fig. S10. Separate channels of Fig. 8a

Supplementary Table 1: Antibodies used in the study

Antibody	Vendor	Catalog Number	Dilution
AcTub	Proteintech	66200-1-Ig	1:2000
AT8	Thermo Fisher	MN1020	1:1000
B-actin	Santa Cruz	SC-47778	1:5000
GSK3β	Cell Signaling	5676	1:2000
HDAC6	Proteintech	12834-1-AP	1:1000
MAP2	Santa Cruz	SC-74421	1:1000
pGSK3β	Millipore-Sigma	05-413	1:2000
pSGK1	Abcam	ab55281	1:3000
SGK1	SAB	32125	1:1000
S214	Thermo Fisher	44-742G	1:1000
TAU	Thermo Fisher	AHB0042	1:1000
Tubulin	Santa Cruz	SC-32293	1:1000
